# Chitosan Nanoparticles Alleviated the Adverse Effects of Sildenafil on the Oxidative Stress Markers and Antioxidant Enzyme Activities in Rats

**DOI:** 10.1155/2023/9944985

**Published:** 2023-01-31

**Authors:** Salah A. Sheweita, Dalia M. Elsayed Alian, Medhat Haroun, Mohamed Ismail Nounou, Ayyub Patel, Labiba El-Khordagui

**Affiliations:** ^1^Department of Clinical Biochemistry, Faculty of Medicine, King Khalid University, Abha, Saudi Arabia; ^2^Department of Biotechnology, Institute of Graduate Studies and Research (IGSR), Alexandria University, Egypt; ^3^Department of Pharmaceutics, Faculty of Pharmacy, Alexandria University, Egypt

## Abstract

Sildenafil (SF) is widely used for erectile dysfunction and other conditions, though with limitations regarding oral absorption and adverse effects. Despite nanotechnological improvements, the effect of nanocarriers on SF hepatotoxicity has not been documented to date. This study aimed at assessing the impact of chitosan nanoparticles either uncoated (CS NPs) or Tween 80-coated (T-CS NPs) on the effects of SF on oxidative stress markers and antioxidant enzyme activities in rats. Test SF-CS NPs prepared by ionic gelation were uniform positively charged nanospheres (diameter 178-215 nm). SF was administered intraperitoneally to male rats (1.5 mg/kg body weight) in free or nanoencapsulated forms as SF-CS NPs and T-SF-CS NPs for 3 weeks. Free SF significantly suppressed the activity of the antioxidant enzymes glutathione S-transferase (GST), glutathione peroxidase (GPx), glutathione reductase (GR), catalase (CAT), and superoxide dismutase (SOD), as well as the levels of glutathione (GSH) and thiobarbituric acid reactive substances (TBARS) as in an indirect measure of free radicals. Interestingly, SF-CS NPs and T-SF-CS-NPs treatments significantly attenuated the inhibitory effects of SF on the activity of these enzymes whereas, GST activity was inhibited. Moreover, the protein expression of GST was downregulated upon treatment of rats with free SF, SF-CS-NPs, and T-SF CS-NPs. In contrast, the activity and protein expression of GPx was induced by SF-CS NPs and T-SF-CS-NPs treatments. The histopathological study showed that SF induced multiple adverse effects on the rat liver architecture which were markedly suppressed particularly by T-SF-CS NPs. In conclusion, chitosan nanoencapsulation of SF counteracted the adverse effects of SF on the activity of antioxidant enzymes and liver architecture. Findings might have significant implications in improving the safety and efficacy of SF treatment of the widely expanding disease conditions.

## 1. Introduction

Sildenafil (SF) is a vasoactive first-generation phosphodiesterase-5 (PDE5) inhibitor approved for the treatment of male erectile dysfunction [1] and pulmonary arterial hypertension [2]. PDE5 inhibitors including sildenafil, tadalafil, and vardenafil act by inhibiting the phosphodiesterase type 5 (PDE5) enzyme present in high concentration in the *corpus cavernosum.* PDE5 specifically breaks down the cyclic guanosine monophosphate (cGMP), responsible for nitric oxide-induced smooth muscle relaxation and vasodilatation [3]. As the PDE5 enzyme is also distributed in many cells throughout the body, PDE5 inhibitors have the potential for wider use in different clinical conditions such as neurodegenerative disorders and brain injuries [4, 5], heart failure [6], and cancer [7].

SF is usually taken orally as film-coated tablets, though with limitations including low oral bioavailability (~40%) as well as delayed onset (30–45 min) and short (half-life ~3 h) duration of action [8]. SF is extensively metabolized in the liver by cytochrome P450 enzymes, mainly CYP3A4 and to a lesser extent CYP2C9 [9], resulting in the loss of a large part of an oral dose [10]. Conversely, SF and other PDE5 inhibitors may affect the activity of antioxidant enzymes such as glutathione S-transferase (GST), glutathione peroxidase (GPx), glutathione reductase (GR), catalase (CAT), and superoxide dismutase (SOD), the levels of thiobarbituric acid reactive substances (TBARS) and glutathione (GSH) as well as the protein expression of different CYPs isozymes in the livers of experimental animals [11–13]. Increased amounts of reactive oxygen species (ROS), such as superoxide (O_2_▪−) and hydroxyl radical (OH▪), and reactive nitrogen species (RNS) as nitric oxide (NO▪) and nitrogen dioxide (NO_2_▪) radicals cause oxidative and nitrosative stress, respectively, when antioxidant systems are suppressed. Such situations play a significant role in the pathophysiology of erectile dysfunction [14] and other disorders [15, 16].

Approaches most adopted to overcome the limitations of oral SF comprise alternative routes of administration to bypass the liver [17, 18] and nanotechnology to improve SF bioavailability, prolong its action and reduce its adverse effects [19, 20]. Despite achievements in this respect, the potential influence of nanocarriers on the hepatic effects of SF has not been documented to date. This initiated our interest in investigating the influence of nanoencapsulation on SF hepatic effects. Chitosan (CS) was selected as the carrier polymer because of its beneficial bioactivities [21] as well as its important role in effective systemic drug delivery and tunable cellular uptake of drugs [22]. Additionally, CS has been shown to protect the liver from hepatocarcinogens and other drug-induced toxicity [23, 24].

This study aimed at assessing the changes induced in the livers of rats upon intraperitoneal administration of SF, SF-CS NPS, and T-SF CS NPs for 21 days both biochemically and histopathologically. Biochemical assessments included the change in the activity of antioxidant enzymes (GST, GR, GPx, SOD, and catalase CAT), the level of GSH and TBARs as an indirect measure of free radicals, and the protein expression of GST and GPx. Biochemical assessments were supported by histopathological examination of changes in the liver of rats.

## 2. Materials and Methods

### 2.1. Materials

Low molecular weight chitosan (50-190 kDa, 75-85% deacetylated) (SC), sildenafil citrate (SF), 5,5′-dithiobis nitrobenzoic acid (DTNB), nicotinamide adenine dinucleotide phosphate (NADPH), bovine serum albumin (BSA), reduced glutathione (GSH), 1-chloro-2,4-dinitrobenzene (CDNB), epinephrine, acrylamide, bisacrylamide, tetramethylethylenediamine (TEMED), cumene hydroperoxide, and tris-HCl were purchased from Sigma Aldrich, Germany. Sodium tripolyphosphate (TPP) was purchased from Loba Chemie, India. Folin-Cioclateu phenol reagent was purchased from Oxford Lab Chem, India. Tween 80, potassium phosphate, trichloroacetic acid (TCA), thiobarbituric acid (TBA), sodium phosphate, hydrogen peroxide (H_2_O_2_), sodium carbonate, magnesium chloride, acetone, sodium hydroxide (NaOH), sodium borate, sodium carbonate (Na_2_CO_3_), copper sulfate (CuSO_4_), Na-K tartrate, sulphosalicylic acid, ammonium persulphate (APS), and sodium dodecyl sulfate (SDS) were purchased from El-Nasr Pharmaceutical Company, Egypt. Primary anti-mouse antibodies for GPx and GST were obtained from Santa Cruz Co., USA.

### 2.2. Preparation of Chitosan-Based Nanoparticles (CS NPs)

Chitosan-based nanoparticles including blank CS NPs, SF-loaded chitosan nanoparticles (SF-CS NPs), and Tween 80-coated SF-loaded CS-NPs (T- SF-CS NPs) were prepared using essentially a TPP ionic gelation method with some modification [25]. In brief, low molecular weight CS was dissolved in an acetic acid solution with magnetic stirring overnight. After pH adjustment to 4.7-4.8 with 20% NaOH, the CS solution was filtered using a 0.45 *μ*m nylon syringe filter. A cold-filtered TPP solution (3 mL) was added to the CS solution (10 mL) in a water bath at 60°C under magnetic stirring for about 10 min and the formed blank SF-CS NPs were separated. To adjust the physical properties of plain CS-NPs, particularly, the particle size and size distribution, a series of preliminary trials based on the preparation of NPs by changing the experimental variables one at a time while keeping other variables constant was undertaken. The variables included the concentration of CS and TPP solutions (0.5 mg/mL vs. 2 mg/mL), the temperature of the CS solution during TPP addition (25°C vs. 60°C) as well and the method of separation of the formed CS NPs (low-speed centrifugation at 3000 rpm for 10 min at ambient temperature *vs* probe sonication for 10 and 20 min).

SF-CS-NPs were prepared by dissolving SF (2 mg/mL) in the CS solution as reported earlier for berberine-loaded CS NPs [26], and the procedure was completed as described above. For the preparation of T-SF-CS-NPs, freshly prepared SF-CS NPs were resuspended in 1% Tween 80 solution and sonicated for 20 min in a water bath sonicator [27].

### 2.3. Characterization of the Chitosan Nanoparticles

#### 2.3.1. Physical Properties

The SF-CS-NPs and T-SF-CS-NPs were characterized for particle size and size distribution expressed as polydispersity index (PDI) by dynamic light scattering (DLS) using Zetasizer Nano ZS Series DTS 1060, Malvern Instruments S.A., Worcestershire, UK at a scattering angle of 90° at 25°C using a 4-mW He–Ne laser at 633 nm. The NP dispersions were suitably diluted 1 : 80 in deionized water and measurements were performed in triplicate. Zeta potential was determined at 25°C in water using a cell voltage of 150 V and 5 mA current.

#### 2.3.2. Transmission Electron Microscopy (TEM)

The morphology of the test NPs was examined by TEM using JEOL, JEM-100 CX Electron Microscope (Tokyo, Japan). Before analysis, NP dispersions were sprayed onto copper grids and stained with 2% *w*/*v* uranyl acetate solution. Shots were taken at ×10 k at 80 kV.

#### 2.3.3. Entrapment Efficiency (EE%)

The SF entrapment efficiency (EE%) was calculated based on the difference between the amounts of entrapped and unentrapped SF. SF-CS-NPs were separated by centrifugation for 30 min at 15000 rpm at 4°C. Unentrapped SF in the supernatant was determined spectrophotometrically at *λ*max 293 nm. EE% was calculated as follows:

(1) $$\begin{array}{c} \text{EE}\%=\frac{\text{Total SF }\left(\text{mg}\right)-\text{Unentrapped SF}\left(\text{mg}\right)}{\text{Total SF }\left(\text{mg}\right)}. \end{array}$$

### 2.4. Treatment of Rats with Sildenafil and Sildenafil-Chitosan Nanoparticles

The study protocol was approved by the Research Ethics Committee of the Medical Research Institute, Alexandria University, and complied with the Guide for the Care and Use of Laboratory Animals of the National Research Council (US), Institute for Laboratory Animal Research. Forty-eight male Wistar rats (average weight of 200 ± 20 g) were obtained from the animal house of the Faculty of Agriculture, Alexandria University. Rats were acclimated for 7 days before the experiment and were provided with a balanced commercial diet. The rats were randomly divided into four groups, 12 rats each. Treatments were administered by a daily intraperitoneal (i.p.) injection for 21 days as follows:

Group 1 (control): 0.3 mL normal physiological saline

Group 2: 1.5 mg/kg SF citrate solution in distilled water (DW)

Group 3: SF-CS NPs (equivalent to 1.5 mg/kg SF)

Group 4: T-SF-CS NPs (equivalent to 1.5 mg/kg SF)

At the end of the treatment period, rats were anesthetized, sacrificed, and their livers were isolated, washed with saline, and kept at -80°C for further biochemical analyses. Liver biopsies for histological examination were kept in 10% formalin.

#### 2.4.1. Assay of Antioxidant Enzymes Activity

The livers of rats were rinsed in cold 0.1 M potassium phosphate buffer (pH 7.4), blotted dry, weighed, and kept on ice. The liver homogenate (33%) was prepared in 3 portions of 0.1 M phosphate buffer (pH 7.4) using a Teflon piston homogenizer on the ice at 4°C. The liver homogenates were then centrifuged for 20 min at 4°C at 11000 rpm to remove intact cell nuclei, mitochondria, and cell debris. The S9 fractions of the livers were stored at -80°C [28].

The GST activity was assayed according to the method of Habig et al. [29]. The calculations were performed using a molar extinction coefficient of 9.6 mM/cm. Under the assay conditions, a unit of enzyme activity was defined as the amount of enzyme that catalyzes the synthesis of 1 mM of CDNB conjugate/mg protein/min. Thiobarbituric acid-reactive substances (TBARS) were detected in the supernatant of S9 fractions [30]. The color intensity of the reactants (TBARS) was measured at 532 nm. An extinction coefficient of 156 000 M^−1^/cm was used in the calculation of the TBARS level. The glutathione levels in the supernatant of liver tissue homogenates were determined using sulfosalicylic acid for protein precipitation and bis-(3-carboxy-4-nitrophenyl)-disulfide for color development [31]. The color intensity at 412 nm was measured using a double-beam spectrophotometer. The activity of glutathione reductase (GR) was determined by monitoring NADPH oxidation at 340 nm in the supernatant of liver tissue homogenates [32]. GR activity was expressed as nmol NADPH oxidized/mg protein/min. The protein concentration was measured using bovine serum albumin as standard [33].

The activity of the SOD enzyme (EC 1.15.1.1) in S9 fractions was measured as reported [34]. The SOD assay was based on the inhibition of epinephrine autoxidation to adrenochrome in an alkaline medium, which is significantly reduced in the presence of SOD. The increase in adrenochrome absorbance was measured spectrophotometrically at 480 nm every 30 s for up to 4 min. The SOD enzyme activity was measured as the quantity of enzyme that prevents epinephrine from being oxidized by 50%, with each 50% inhibition equaling one unit (1 U/g tissue). The catalase (CAT) (EC1.11.1.6) activity was measured by the method of Beers and Sizer, 1952. The assay is based on the spectrophotometric measurement of H_2_O_2_ decomposition at 240 nm. A known volume (2.5 mL) of H_2_O_2_ buffer (0.15 M sodium-potassium phosphate buffer pH 7.0) and 50 *μ*L of S9 enzyme source were used in the experiment. The absorbance was determined spectrophotometrically at 240 nm after 20 and 40 s intervals against blank. The CAT enzyme activity was expressed as unit/mg protein. One unit of CAT is equal to one nmol H_2_O_2_/mg protein/min.

#### 2.4.2. Western Blotting and Detection of Immobilized Proteins

Aliquots (100 *μ*L) of the S9 fraction from each rat (10 rats per group) were pooled and used to examine the protein expression of GST and GPx. Each group's pooled microsomal proteins (40 *μ*L) were combined with sample application buffer (SAB) and heated for 3 min before loading on a 10% SDS-polyacrylamide gel. Proteins were transferred to nitrocellulose membranes using a semidry transblotter after electrophoresis. They were washed three times with TBS buffer pH 7.3 (8 g NaCl, 0.2 g KCl, and 3 g Tris-base/L) for 10 min after completing the transblotting of proteins on membranes. The membranes were then rinsed in Tris-HCL buffer saline (T-TBS) buffer containing 0.1% Tween 20 for 5 min and then in TBS buffer twice for 10 min after being incubated with 5% fat-free dry milk-TBS buffer for 1 h at room temperature. The membranes were then incubated for 2 h with primary antibodies for anti-GST, and anti-GPx at a dilution of 1 : 1000 before being washed twice with Tween 80-TBS (0.2 ml Tween 20/L TBS) for 20 min and TBS for 15 min. After incubation with anti-mouse horseradish peroxidase-conjugated secondary antibody at a dilution of 1 : 7000 in TBS, the membranes were washed twice with Tween 80-TBS for 15 min and then twice with TBS for 15 min. The protein expression of various isozymes was identified using an ECL kit and X-ray film. The intensity of the bands was determined using the Quantity One Software Program (version 4.6.9, Bio-Rad Co., California, USA).

#### 2.4.3. Histopathological Examination

Small sections of both liver tissues from each rat in each treatment were preserved in a 10% formaldehyde solution, embedded in paraffin wax, and sectioned with a microtome into 3 *μ*m-thick sections which were stained with Hematoxylin and Eosin (H&E) and examined by light microscopy (Olympus BX 50, Japan) to identify histopathological changes [35].

### 2.5. Statistical Analysis

The results were presented as means ± SE. A one-way analysis of variance was used to calculate the differences between groups (ANOVA) using the SPSS Statistics Program version 20. Differences between groups were considered significant at *P* < 0.05.

## 3. Results

### 3.1. Characteristics of Chitosan-Based Nanoparticles

**Figure 1:**
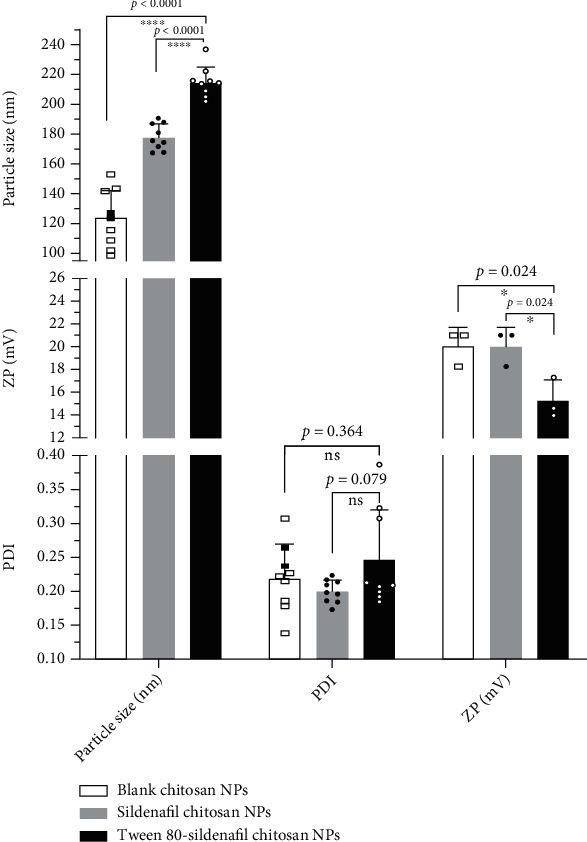
Physical properties of blank chitosan NPs, sildenafil chitosan NPs, and Tween 80-coated sildenafil chitosan NPs.

Chitosan-based NPs prepared using a 2 mg/mL concentration of CS and TPP, a CS: TPP ratio of 3: 1, and probe sonication on ice for 10 min displayed desired physical properties. The physical properties of blank CS NPs, SF-CS NPs, and T-SF-CS NPs are shown in Figure 1. Blank CS NPs had a mean size of 124.2 ± 20.2 nm, a mean polydispersity index (PDI) of 0.221 ± 0.05, and a mean zeta potential of 18.0 ± 2.0 mV. SF-loaded CS NPs (SF-CS NPs) showed a significantly (*P* < 0.0001) larger mean size (178 ± 12.1 nm) with no significant changes in PDI and ZP. Tween 80-coated SF-CS NPs (T- SF-CS NPs) exhibited a further significant increase in size (215.3 ± 10.5 nm) relative to SF-Cs NPs (*P* < 0.0001) in addition to a small increase in PDI and a reduction in ZP.

**Figure 2:**
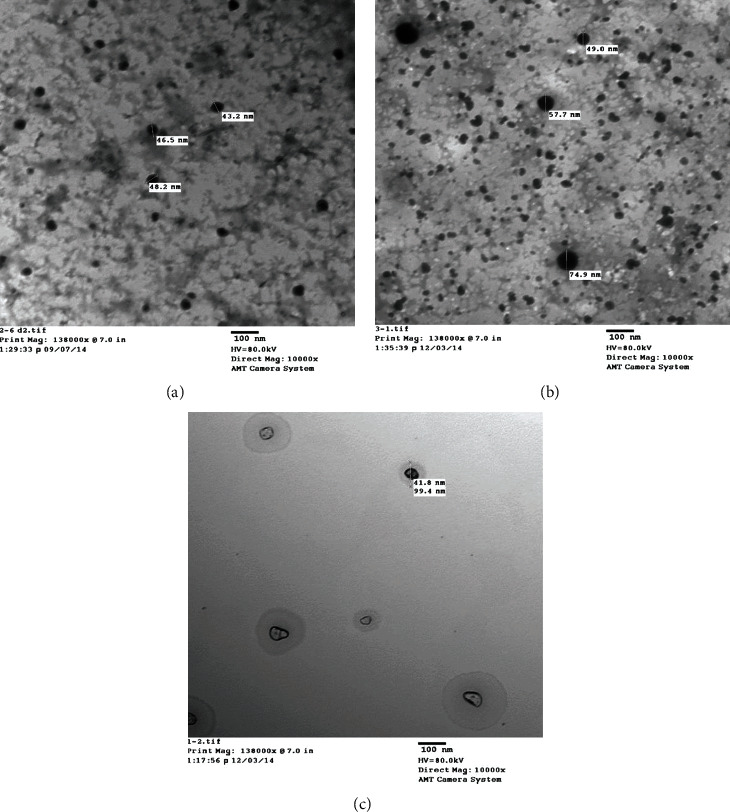
TEM of Blank chitosan nanoparticles (CS NPs) (a). Sildenafil-loaded CS NPs (SF-CS NPs) (b). Tween 80-coated SF-CS NPs (T-SF-CS NPs) (c).

TEM imaging of the test CS-based NPs (Figure 2) indicated that blank CS NPs (Figure 2(a)) were uniform nonaggregated nanospheres with a size ranging from 43.2 to 48.2 nm. SF-CS NPs were also spherical but slightly larger (Figure 2(b)). Tween 80 surface coating led to a further increase in the size of SF-CS NPs which appeared surrounded by a clear zone (Figure 2(c)). The mean EE of SF was 27.63 ± 1.25%.

### 3.2. Hepatic Biochemical Effects of Chitosan-Based Nanoparticles

**Figure 3:**
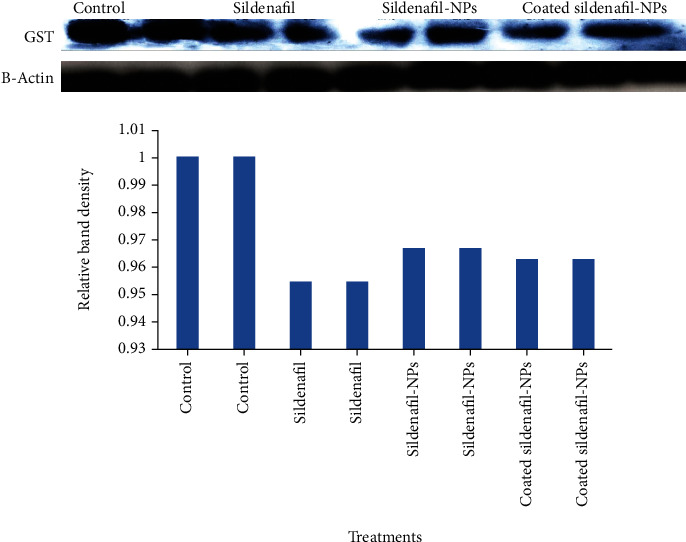
Western blot analysis of the effect of sildenafil, sildenafil-chitosan NPs, and Tween 80-sildenafil-chitosan NPs on the protein expression of GST and its relative band density.

As shown in Table 1, SF administration to rats drastically reduced the levels of GSH and TBARs. Compared with the SF-treated group, SF-CS NPs and T-SF-CS NPs treatments considerably boosted GSH levels, though normal levels were not recovered (Table 1). Similarly, both SF-CS NPs attenuated the inhibitory effects of SF on the TBARS level which was not significantly different from the control level following treatment with T-SF-CS NPs. Western immunoblotting data (Figure 3) demonstrated that the protein expression of GST was downregulated which may account for the effects of SF, SF-CS-NPs, and T-SF-CS-NPs treatments on GST activity (Figure 3).

**Figure 4:**
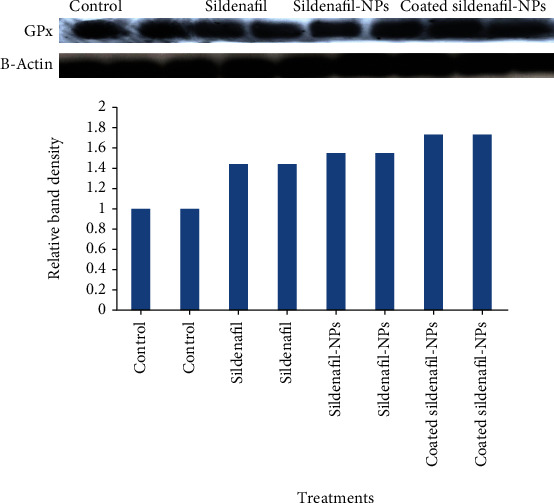
Western blot analysis of the effect of Sildenafil; Sildenafil-Chitosan NPs and Tween 80-Sildenafil-Chitosan NPs on the protein expression of GPx and its relative band density.

The effect of SF, SF-CS NPs, and T-SF-CS NPs treatments on the activity of the antioxidant enzymes glutathione reductase (GR), catalase (CAT), glutathione peroxidase (GPx), and superoxide dismutase (SOD) is shown in Table 1. In contrast to a significant increase in GPx activity, free SF therapy significantly reduced the activity of GR, CAT, and SOD. Western immunoblotting findings showing an increase in GPx protein expression after SF-CS NPs and T-SF-CS NPs treatments verified the results of GPx activity (Figure 4). It is interesting to note that CAT, GR, GPx, and SOD activities were dramatically increased by SF-CS NPs and T-SF-CS NPs treatments relative to either SF and/or the control groups (Table 1).

### 3.3. Histopathological Examination

**Figure 5:**
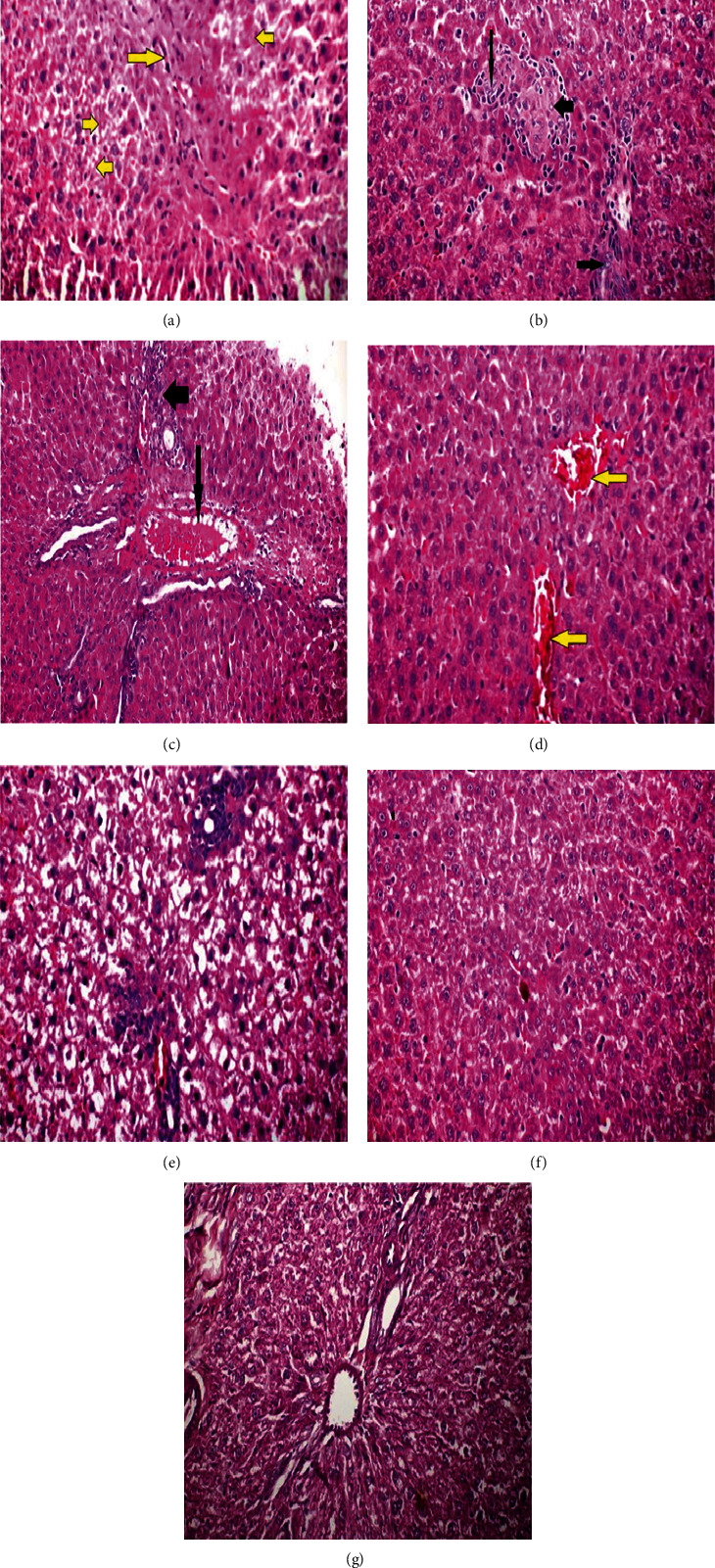
Photomicrographs of liver sections from rats treated for 21 days with: (a–d) sildenafil; (e) uncoated SF chitosan nanoparticles (SF-CS NPs); (f) Tween 80-coated chitosan nanoparticles (T-SF-CS NPs) in comparison with (g) sections from untreated control rats. Adverse hepatic effects of sildenafil included (a) necrosis of the hepatic cells with loss of nuclei and destruction of the normal hepatic cells (long arrow) and fibrosis of hepatocytes (short arrows); (b) regeneration of hepatic cells with oval cells hyperplasia (long arrows) and regeneration of binucleated hepatic cells with the vesicular body (short arrow); (c) hemolysis of the blood in the portal veins (long arrow) associated with inflammatory cells with fibrosis in the portal areas (short arrow); and (d) dilation of central veins engorged with blood (arrows). SF-CS NPs treatment (e) resulted in white fluffy cytoplasm surrounding the central nucleus of hepatocytes and aggregation of inflammatory cells surrounding the central vein and portal region ballooned in size. Sections for the rat group treated with T-SF-CS NPs (f) showed a liver architecture and cells close to those of untreated normal control rats (g), indicating protection of the liver against the harmful effects of SF and CS NPs. Hematoxylin and eosin (H&E) stained sections at 400×.

Histopathological examination (Figures 5(a)–5(g)) demonstrated changes in the architecture of liver tissues after treatment with SF, SF-CS NPs, and T-SF-CS NPs compared with the control liver. Liver sections from SF-treated rats (Figures 5(a)–5(d)) showed several hepatotoxic effects including necrosis of hepatic cells with loss of nuclei and fibrosis (Figure 5(a)), regeneration of hepatic cells with oval cell hyperplasia (long arrows) and binucleated cells (short arrows) (Figure 5(b)), hemolysis of blood in the portal veins (long arrows) and inflammatory cells with fibrosis in the portal areas (short arrows) (Figure 5(c)) in addition to dilation of central veins that were engorged with blood (Figure 5(d)). Chitosan nanoencapsulation of SF led to different hepatic changes including ballooning degeneration of hepatocytes with white fluffy cytoplasm surrounding the central nucleus and accumulation of inflammatory cells around the central vein and portal area (Figure 5(e)). Such changes were markedly suppressed by T-SF-CS NPs treatment (Figure 5(f)) which protected the normal liver architecture and hepatic cells. The histological characteristics of liver sections of the T-SF-CS NPs-treated group were close to those of the normal control section (Figure 5(g)).

## 4. Discussion

### 4.1. Chitosan-Based Nanoparticles

The CS NPs under study (Blank CS NPs, SF-CS NPs, and T-SF-CS NPs) exhibited accepted mean size (range 124 to 215 nm) and mean polydispersity index (PDI range 0.20–0.25) indicative of nanosize and narrow particle size distribution [36]. The test NPs displayed a positive surface charge as indicated by a zeta potential range of 15.4-20 mV. This is of importance to maintaining the colloidal stability of the NPs. The relatively high ZP of CS NPs is attributed to the protonation of CS amino groups [37]. The mean size of blank CS NPs was increased by SF loading, an observation consistent with literature reports [38, 39]. The further significant increase in mean SF-CS NPs size by Tween 80 coating of SF-CS NPs can be ascribed to adherence of the Tween 80 surfactant molecules to the hydrophilic surface of CS NPs, probably increasing their hydrodynamic radii. Tween 80 coating also slightly decreased the ZP of NPs, as a result of the partial concealing of the surface positive charge of CS NPs CS NPs [40].

TEM imaging provided evidence for the uniformity of shape and size of the three test CS NPs and revealed a clear zone around the T-SF-CS NPs. Despite differences in absolute values, the particle size of the three nanoformulations shown by TEM was in agreement with that determined by DLS. The smaller size of NPs generally observed in TEM images can be explained by the dehydration of NPs during sample preparation [41]. The colloidal properties and morphological traits of the three CS NP formulations were generally appropriate for drug delivery applications [22].

### 4.2. Effect of Chitosan-Based Nanoparticles on the Activity of Antioxidant Enzymes

Glutathione (GSH) and glutathione S-transferase enzyme (GST) play a major role in drug conjugation and detoxification, affecting the efficacy of several chemotherapeutic and alkylating drugs [42]. Accordingly, any organ with low GSH levels and inhibited GST activity is more sensitive to alkylating agents, whereas those with high GSH levels and induced GST activity are more protected and resistant to these agents [43–45]. In the present study, GSH levels and GST activity were significantly decreased after the treatment of rats with SF. Although SF-CS NPs and T-SF CS NPs alleviated the inhibitory effect of SF, normal levels of GSH levels and GST activity were not restored. Data from western immunoblotting confirmed the results of GST activity by demonstrating that all treatments decreased the protein expression of GST in comparison to the control group. This might imply the contribution of CS to the SF-induced suppression of GST protein expression. In support of this assumption, it has been reported that rats fed chitosan also showed lower liver activity of GST [46]. As reported previously, SF therapy reduced GSH levels and inhibited GST activity in the liver tissues of rats [11] and human red blood cells [47]. Possible conversion of reduced glutathione (GSH) into its oxidized form as a result of SF, SF-CS NPs, and T-SF-CS NPs treatments may account for the decline in GSH levels. As oxidized glutathione may be reverted into its reduced form by the enzyme GR, the reduction of GR by SF treatment as reported earlier [11] might account for the findings. While SF-CS-NPs were unable to reverse the SF-induced suppression of GR activity, T-SF-CS NPs restored the activity to its control level.

It has been shown that when GST activity is suppressed and GSH levels are low, the epoxides of well-known chemical carcinogens bind to DNA and other macromolecules more covalently [43, 44, 48] which enhances hepatocarcinogenesis. This entails that SF therapy may increase hepatotoxicity as a result of decreasing GSH levels and GST activity. Interestingly, T-SF CS NPs enhanced both GSH levels and GST activity, suggesting possible liver protection from the hazardous effects of chemical substances produced endogenously or upon exposure to exogenous sources.

Reactive oxygen species (ROS) have been implicated in the induction of oxidative stress [49]. According to data obtained in the present study, the levels of TBARS were significantly lower in the groups treated with free SF, SF CS-NPs, and T-SF CS NPs compared with the control group. Similarly, earlier research demonstrated that SF treatment reduced free radical levels, resulting in subsequent inhibition of lipid peroxidation [50]. In addition, GPx and CAT enzymes are involved in the termination reaction of the ROS pathway. The data of the present study revealed that there was a significant increase in GPx activity and upregulation of its protein expression after treatment of rats with SF CS-NPs and T-SF CS NPs relative to the control group. CAT seems to be the main regulator of hydrogen peroxide metabolism [51]. SF-CS-NPs- and T-SF-CS NPs-treated groups significantly increased CAT and SOD activity in comparison with the control group. As a result, stimulation of GPx and CAT which are acting as radical ions quenchers could explain the mechanism of reduction of free radical levels in SF CS-NPs- and T-SF CS NPs-treated groups.

### 4.3. Histopathological Examination

The results of histopathological examination revealed multiple SF-induced hepatotoxic effects including necrosis of hepatic cells with loss of nuclei and fibrosis; regeneration of hepatic cells with oval cell hyperplasia and binucleated cells; hemolysis of blood in the portal veins and inflammatory cells with fibrosis in the portal areas in addition to dilation of central veins which appeared engorged with blood. These findings raise concerns regarding the safety of SF treatment of different diseases and urge the need for strategies to reduce the adverse effects of SF on the liver. As demonstrated in the present study, nanoencapsulation of SF might influence its hepatotoxic profile. Encapsulation of SF by CS NPs modified the SF-induced histopathological changes which were manifested as an accumulation of inflammatory cells in the central vein and portal area with degeneration of hepatocytes. Likewise, CS NPs with a size of 200 nm were reported to induce severe defects in zebrafish embryos, including a twisted spine, pericardial edema, and an opaque yolk, supporting our findings [52]. In addition, embryos exposed to CS NPs had a higher rate of cell death, higher levels of ROS, and overexpression of heat shock protein 70, verifying the induction of physiological stress in zebrafish by CS NPs [52]. Intriguingly, the Tween 80 coating of SF-CS NPs significantly protected the liver architecture and hepatic cells from the harmful effects caused by SF and uncoated CS NPs as verified by the close-to-normal architecture of liver sections from T-SF-CS NPs-treated rats. In comparison with liver sections of the SF-CS NPs-treated group, sections of T-SF-CS NPs-treated and untreated control groups showed a network of hepatic strands made up of almost normal hepatocytes structures. As such, T-SF-CS NPs were effective in preventing the deleterious hepatic histopathological changes induced by SF and CS NPs.

## 5. Conclusion

A nanotechnological approach was explored to suppress the hepatic adverse effects and improve the safety of sildenafil. The drug was encapsulated in uncoated and Tween 80-coated chitosan nanoparticles characterized for colloidal properties and drug entrapment efficiency. The adverse hepatic effects of sildenafil manifested as suppression of antioxidant enzymes activity and multiple deleterious changes in liver tissues of rats following a 21-day-intraperitoneal treatment, were attenuated particularly by the Tween 80-coated nanoparticles. Findings are of significance regarding the improvement of the safety of sildenafil therapy, a drug currently under active investigation for safer treatment of erectile dysfunction, pulmonary arterial hypertension, and an expanding list of disease conditions.

## Figures and Tables

**Table 1:** Effect of sildenafil, sildenafil-NPs, and Tween 80-coated sildenafil-NPs on the activity of antioxidant enzymes.

Parameter	Control	Sildenafil	Sildenafil NPs	Tween 80-sildenafil NPs
GSH levels (*μ*mol/g liver)	5.00^a^ ± 0.25	2.98^b^ ± 0.14	3.00^b^ ± 0.16	3.51^b^ ± 0.24
TBARS (*μ*mol/g liver)	0.47^a^ ± 0.018	0.33^b^ ± 0.02	0.34^b^ ± 0.013	0.38^**a**^ ± 0.017
GST activity (unit/mg)	3.13^a^ ± 0.15	1.28^b^ ± 0.16	2.642^b^ ± 0.14	2.475^b^ ± 0.07
GR activity (nmol oxidized NADPH/mg protein/min)	35.00^a^ ± 0.85	22.15^b^ ± 1.28	38.94^a^ ± 1.22	48.28^a^ ± 1.56
GPx activity [unit/ mg protein]	14.25^c^ ± 1.22	19.31^b^ ± 1.14	23.80a ± 0.92	26.70^a^ ± 1.39
Catalase activity (*μ*mol H_2_O_2_/mg protein/min)	27.15^b^ ± 0.58	17.75^c^ ± 0.53	29.52^c^ ± 0.67	39.60^a^ ± 1.04
SOD activity (unit/mg protein)	12.00^b^ ± 0.55	8.33^b^ ± 0.48	16.40^a^ ± 0.53	22.61^b^ ± 0.35

NPs are chitosan nanoparticles. All values are presented as means ± SE. Means with different superscripts are statistically significant, whereas means with similar superscripts are statistically non-significant (*P* < 0.05).

## Data Availability

All available data are included in the MS.

## Abbreviations

SF: Sildenafil
SF CS-NPs: Sildenafil chitosan nanoparticles
T-SF CS NPs: Tween 80-coated sildenafil chitosan nanoparticles
GPx: Glutathione peroxidase
GST: Glutathione S-transferase
GSH: Glutathione
CAT: Catalase
SOD: Superoxide dismutase.
